# Qualitative Review of National Nutrition Surveillance Systems in the Eastern Mediterranean Region

**DOI:** 10.3390/nu15173689

**Published:** 2023-08-23

**Authors:** Ayoub Al Jawaldeh, Ola El Hajj Hassan, Abdul Baseer Qureshi, Ferima Coulibaly Zerbo, Shafekah Alahnoumy, Mahmoud Bozo, Mousa Al-Halaika, Mushary H. Al-Dakheel, Lamya Alhamdan, Sahibzada Azhar Mujib, Laila El Ammari, Hassan Aguenaou, Nawal Alqaoud, Salima Almaamari, Saleh Alshamkhi, Fekri Dureab

**Affiliations:** 1 World Health Organization (WHO), Regional Office for the Eastern Mediterranean, Cairo 7608, Egypt; 2 Heidelberg Institute of Global Health, Hospital University Heidelberg, 69120 Heidelberg, Germany; 3 World Health Organization (WHO), Khartoum 2234, Sudan; 4 World Health Organization (WHO), Sana’a 543, Yemen; 5 World Health Organization (WHO), Damascus 3946, Syria; 6 Nutrition Department, Ministry of Health, Ramallah 4284, Palestine; 7 Nutrition Department, Ministry of Health, Riyadh 11176, Saudi Arabia; 8 Nutrition Department, Ministry of Health, Rabat 335, Morocco; 9 Joint Research Unit in Nutrition and Food, RDC-Nutrition AFRA/IAEA, Ibn Tofaïl University-CNESTEN, Rabat-Kénitra 242, Morocco; 10 Food and Nutrition Administration, Ministry of Health, Kuwait City 13001, Kuwait; 11 Nutrition Department, Ministry of Health, Muscat 393, Oman; 12 Institute of Research for International Assistance, Akkon Hochschule, 12099 Berlin, Germany

**Keywords:** nutrition assessment, surveillance, Arab countries, malnutrition, emergency response

## Abstract

The World Food Conference in 1974 emphasized the significance of establishing global nutrition surveillance to monitor and address nutritional challenges effectively. However, many countries, especially in the EMRO region, continue to encounter substantial difficulties in regularly generating disaggregated data on nutrition. The current study aimed to review the existing nutrition surveillance systems in the region and to identify their strengths and weaknesses, as well as the challenges they face in functioning optimally. Methods: This study focused on the functional nutrition surveillance systems in eight Arab countries; namely Kuwait, Morocco, Oman, Palestine, Saudi Arabia, Sudan, Syria, and Yemen. The study’s analysis involved utilizing primary data collected from both published and unpublished reports. Additionally, a structured checklist was employed to gather information from all countries involved in the study. Furthermore, interviews were conducted with the EMRO offices to gain deeper insights into the challenges, if any, that these nutrition surveillance systems face in functioning optimally. Results: All countries use health facilities as a basic source of data for their nutrition surveillance, some countries triangulate their nutrition surveillance reports with data from other sources of information such as community or school surveys. Identified nutrition surveillance approaches are closely split between those who operate in stable settings and use routine health information systems (Morocco, Saudi Arabia, Oman, and Kuwait) and other countries that operate in fragile settings; for example, Yemen, Syria, Palestine, and Sudan struggle to provide early warning reports for rapid nutritional responses. Conclusions: Nutrition surveillance systems that utilize existing health information systems are the most sustained in the EMRO region. However, by integrating data from multiple sources, such as health facilities, surveys, and population censuses, countries can provide a holistic view of the nutritional situation, enhance their response to any emergency, and can leverage the infrastructure and resources already in place for health data collection and reporting. Collaboration between countries in the region through sharing experiences and success stories is important in order to reach a standardized system that can be implemented in different settings.

## 1. Introduction

Achieving the Global Nutrition Targets and Sustainable Development Goals (SDGs) by the year 2030 in a precise and timely manner is of utmost importance for both national and global progress. However, the realization of these commitments faces significant challenges due to the scarcity of regular data [1]. The nutrition surveillance system acts as a mechanism designed to translate food and nutrition data into actionable measures by formulating, modifying, and implementing a country’s food and nutrition policy [2]. The data collected through surveillance on the nutritional status of populations serves as a fundamental foundation for the development and planning of policies, as well as the management of programs aimed at enhancing nutrition. Additionally, the system facilitates the establishment of an early warning system to monitor the food consumption patterns and nutritional status of the population [2]. This proactive approach allows for timely interventions and effective measures to address any potential issues related to nutrition and food security [2].

Five different approaches can be used for nutrition surveillance. The first method of data collection is through large-scale food and nutrition surveys, in which the surveillance system records all large national surveys about health, food, and nutrition (such as the Demographic Health Survey). Another method for data collection can include repeated small-scale surveys, which are population-based surveys. These surveys measure the type, severity, and extent of malnutrition and its causes among a representative sample of the population. Also, they help policymakers prioritize areas and populations at risk. A third method of nutrition surveillance is sentinel site surveillance. This method implicates a limited number of sites to detect trends in the population’s overall well-being. Various indicators are monitored including nutritional status, morbidity, dietary issues, coping strategies, and food security. A fourth method of nutritional surveillance includes school census data. This method comprises occasional nutritional assessment in schools. The aim behind school data is to identify children at risk of poor health or malnutrition and low socioeconomic status on the one hand and the main causes of obesity on the other hand. Results from school censuses can be used to target school feeding programs and support food-based strategies. Finally, continuous growth monitoring is also a method for data collection that helps identify any faltering growth issues in children and thus helps correct the problem directly. In emergencies, rapid data and information are crucial to highlight those groups at risk and in need of the rapid assistance used as early warning and measuring trends [2].

The Eastern Mediterranean Region is home to a diverse population of 680 million people across 22 countries, each facing varied socioeconomic statuses and health challenges. A significant portion, approximately 76 million people in the region, are directly or indirectly affected by conflicts, natural disasters, and environmental threats [3]. Moreover, the region hosts over 30 million displaced individuals, primarily due to ongoing conflicts. These crises have profound implications for the well-being of populations and health systems. As a result, food insecurity has surged during these emergencies, with Yemen experiencing the most severe food crisis in the world due to its conflict [3].

The diversity within the region poses challenges in terms of improving the health and nutritional status of its populations. Furthermore, data collection and analysis concerning nutrition remain significant hurdles in the Eastern Mediterranean Region. Only a few countries in the last decade have managed to develop nutrition surveillance systems (NSS) and generate evidence for program development [3]. For example, the Kuwaiti Nutrition Surveillance System serves as an excellent example of a successful system that effectively monitors the health and nutritional status of the Kuwaiti population. This system has demonstrated its capability to provide reliable and sustainable data on the nutritional status of people both at the national and international levels [4,5]. Another example is Sudan which has a national nutrition system that focuses on crisis management and uses information for advocacy and fundraising [4].

To address these challenges, the Regional Strategy on Nutrition for the Eastern Mediterranean Region (2020–2030) was established. This strategy aims to support member states in establishing or strengthening their food and nutrition surveillance systems. The main objectives are to monitor the nutrition situation regularly, provide essential information on key nutrition indicators for vulnerable groups, and identify the socio-economic determinants influencing health and nutrition [3]. By implementing this strategy and improving nutrition data collection and analysis, the region can better understand the nutritional needs of its populations and design targeted interventions to address food insecurity and malnutrition among the most vulnerable groups.

### The Rationale of the Study

Establishing nutrition surveillance systems is a crucial aspect of ensuring that governments can effectively safeguard the nutritional status of their populations [4]. However, in the Middle East Region, there is a significant lack of published information regarding the designs of nutrition surveillance systems that have been adopted and the challenges faced during their development. This knowledge gap highlights the need for a comprehensive study to evaluate and develop potential nutrition surveillance systems tailored for the Middle East.

The importance of conducting such a study lies in its potential to generate valuable evidence. By reviewing and comparing existing systems in the Eastern Mediterranean Region (EMRO), researchers can gain insights into the strengths and weaknesses of these systems, enabling a better understanding of how they can function optimally. Therefore, this study aims to review and compare the existing systems found in the EMRO region and to access the strengths and weaknesses that these systems might be facing to function fully. Based on the findings, the study can contribute to the advancement of surveillance strategies that effectively monitor nutritional trends in the region and address nutritional issues in the region. Moreover, the study’s outcomes may foster collaborations among stakeholders and contribute to the formulation of new policies aimed at enhancing nutrition surveillance and nutritional well-being in the region.

## 2. Materials and Methods

### 2.1. Study Design and Setting

This is a qualitative study using key informant interviews, checklist, and literature reviews as the main approaches used to collect information on national nutrition surveillance systems in the EMRO region. This study performed a comprehensive literature review using multiple search engines, namely Google, Google Scholar, and PubMed. The search was focused on the keyword “nutrition surveillance”, limited to the period from 2003 to 2023 and restricted to English-language documents, a total of 175 peer and grey-reviewed papers were initially identified. After conducting title screening and excluding irrelevant papers, ultimately 19 relevant documents were included in the analysis.

**Figure 1:**
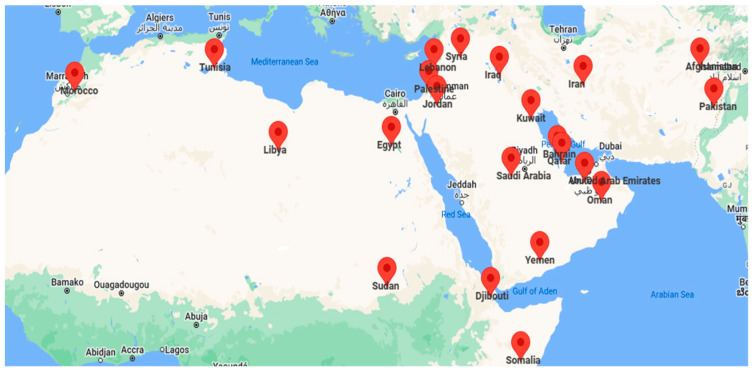
Countries of the Eastern Mediterranean Region [6].

Among the 22 countries found in the region that were contacted to participate in the study, only 8 countries accepted to take part and undergo an interview to understand country-specific nutrition surveillance systems. These countries were namely Kuwait, Saudi Arabia, Oman, Morocco, Sudan, Yemen, Syria, and Palestine. To have an equal and participatory role at all levels of the research, the regional Nutrition Advisor of EMRO revised the concept note and approved the study, and guided approaches to address the challenges in the region. Figure 1 shows the countries in the Eastern Mediterranean Region.

### 2.2. Sampling and Data Collection

**Figure 2:**
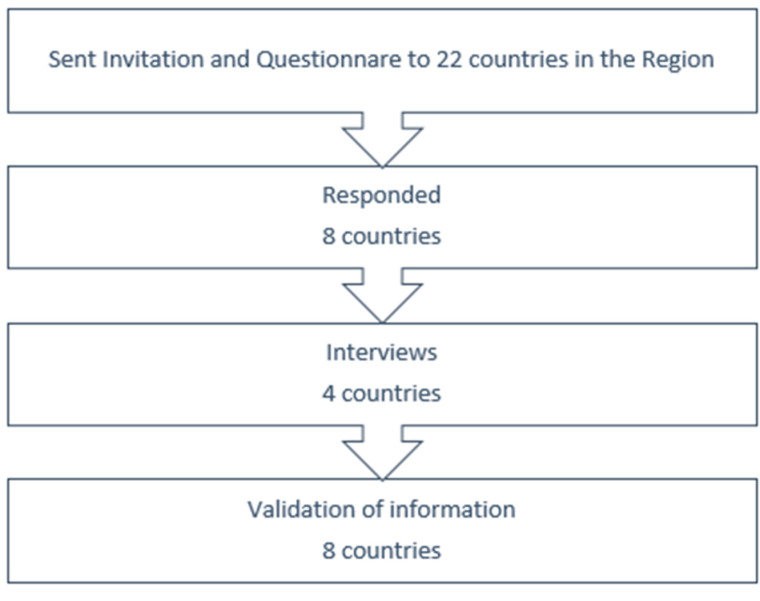
Flowchart of data collection.

Primarily, published and unpublished literature, including nutrition surveillance bulletins from the included states and other reports from UN agencies and the World Health Organization, were reviewed. Moreover, key informants were identified using a snowball approach, including relevant staff working in the nutrition programs in the World Health Organization country offices who were asked to identify other relevant key informants in collaboration with ministries in participating countries. Data was collected using a structured checklist that was developed using a pre-hand specified matrix adopted and published by Friedman et al. [4]. This matrix was reviewed and adopted according to the relevance of the EMRO situation and was approved by the EMRO country office director and country offices before it was used. The matrix reveals topics on the following: type of surveillance, type of data, methods of data collection, and evaluation methods of the surveillance system. The checklist including the matrix was sent to all the 22 countries in the region. Later, four follow-up interviews were conducted online with the nutrition departments in Yemen, Sudan, Syria, and Morocco to clarify several aspects of the checklist to understand the structure of the nutrition surveillance in those countries, and to discuss the challenges facing them to run a well-functioning nutrition surveillance system. Finally, the findings were shared via emails with all included countries for validation. (See the flow chart in Figure 2). All data was collected from January to May 2021.

### 2.3. Data Analysis

The primary data were analyzed manually. Interviews were organized coded and themed accordingly.

### 2.4. Ethical Approval

The official permission to collect data was sort by the Ministry of Health of the identified countries before the start of the work; all countries in this study received an official request to revise the tool and share their consent to provide the required data about the nutrition surveillance system. Participant consent was waived due to this being a program data analysis and the patient details were not included in the analysis.

## 3. Results

Out of 22 identified countries in the EMRO region, 8 countries namely Morocco, Oman, Sudan, Palestine, Kuwait, Syria, Yemen, and Saudi Arabia took part in this study showing a response rate of 38%. The rest of the countries did not respond to our request and showed no interest in participating in this study.

### 3.1. Main Objectives of the Nutrition Surveillance Systems

The key objectives of the nutrition surveillance systems vary, showing similarities and differences among different countries. The main difference is that systems operate in two different settings, stable and fragile settings. Morocco, Saudi Arabia, Oman, and Kuwait have systems that function in stable and secure settings whereas other countries such as Yemen, Syria, Palestine, and Sudan are weakened due to crises and operate in fragile settings. However, all identified nutrition surveillance systems aim to describe the nutritional status of their population and identify malnutrition cases, with particular reference to defined vulnerable subgroups (children under 5 years and pregnant women) as targeted groups. Also, all countries provide regular information and most countries identify trends reflecting the key nutrition indicators on different targets. The countries with stable settings such as the Sultanate of Oman, the Kingdom of Saudi Arabia, and Kuwait target all age groups including those who have special conditions such as chronic diseases, and pregnant and lactating women, whereas the other systems that operate mainly in fragile settings such as Sudan, Yemen, Syria and Palestine focus on the most vulnerable groups, mainly children under 5 years. Most countries use surveillance data to promote health and nutrition policies. For instance, Sudan promotes neural tube defects. Morocco, Kuwait, Palestine, Sudan, and Yemen aim through their systems to provide information on the contributing factors of malnutrition. Furthermore, systems in fragile settings strive to provide early warning reports for potential nutritional actions.

### 3.2. Data Collection Approaches Used by the Nutrition Surveillance Systems

There are different approaches used by the Arab countries in EMRO for collecting data. All eight countries used health facilities as a basic source of data for their nutrition surveillance (Table 1). However, Sudan for example depends on other sources for nutrition surveillance, in which they rely on reports from all of the nutrition centers in the country. Yemen selects health facilities, as sentinel sites, to sensitize the nutrition situation as an early warning system. Furthermore, some countries triangulate their nutrition surveillance reports with data from other sources of information. As an example, Sudan incorporates a middle upper arm circumference (MUAC) community survey provided by some states every six months and anemia indicators from reproductive health reports. Palestine, Morocco, Kuwait, and Oman complement their health facility data from community or school surveys and incorporate the results into the nutrition surveillance annual report (see Table 1).

### 3.3. Indicators Measured by the Nutrition Surveillance Systems

All countries collect anthropometric measures, mainly height and weight, BMI, and Z-score to identify nutritional status. Some countries, such as Yemen and Sudan use middle upper arm circumference (MUAC) to identify the nutritional status such as wasting. All identified systems also collect biochemical data to track micronutrient deficiency (specifically hemoglobin and hematocrit to detect anemia) focusing on pregnant women and children under 5 years. In addition to children and pregnant women, Saudi Arabia focuses on the population with chronic diseases. Palestine and Kuwait track the following indicators for children 0–59 months of age: measles and vitamin A supplementation in the past 6 months, in addition to a diverse range of nutrition-related contributing factors, including dietary habits and food intake. Clinical signs such as edema are reported to be collected from Sudan, Syria, and Yemen. Finally, all countries consider breastfeeding as an important indicator for the health of children and report it together with indicators (see Table 2).

### 3.4. Characteristics of the Survey Tool in Different Countries: Sampling and Geographic Coverage

The sample design of the surveys varied between the systems of the eight different countries. Health facility-based data that are collected by all the countries are collected with no population sampling because they include all care seekers who visit health centers. Sampling is applicable for those countries that conduct surveys. Morocco specifically uses population projection, which is based on the population census as an estimate for their sample. Saudi Arabia undergoes stratified cluster sampling (rural/urban), and pilot survey of registered health data RHDs (Sampling as described in Geneva WHO Jelliffe1966 and AM J Public Health Christakis 1973). In Palestine, the sampling is conducted in cooperation with the Palestinian Central Bureau of Statistics and according to the prevalence of anemia. Kuwait collected the sample size based on the total number of the national population. Finally, in Oman, a National Nutrition Survey in 2017 was conducted, in which a sample of households was selected from among those Omani households selected for the WHO STEPWISE survey of chronic diseases and risk factors. Sample sizes were sufficient to make governorate-specific estimates of many nutrition-related conditions.

Geographic coverage of the surveillance systems also varies to some extent. Systems operating in stable conditions have representative data nearly from all national regions with some specific variations; for instance, Kuwait covers the national people who are almost 33% of the whole population who live in the country. The systems in fragile settings target subnational areas of height vulnerability. Palestine collects data from all governorates including 58 antenatal clinics, 71 mother-child health centers, and 86 schools. Sudan collects data from all nutrition centers in the country. Syria covers 13 out of 14 governorates, and Yemen covers 21 governorates out of 22.

The frequency of data collection varies greatly among the surveillance systems. In Morocco, Oman, and Kuwait the data collection occurs on a rolling basis throughout the year; in Saudi Arabia and Sudan data are collected quarterly while Syria, Yemen, and Palestine collect data every month. However, Palestine and Oman complement data collection from schools and universities every year and Morocco includes nutrition data from school and community surveys every five years. The reporting system does not always reflect the same period of data collection, for instance; Saudi Arabia collects data quarterly while the report is produced annually. Similarly, Oman reports annually; however, data is gathered monthly. Kuwait, Palestine, and Morocco report annually, whereas others provide monthly reports such as Syria and Yemen, while Sudan provides reports in a quarterly sequence.

### 3.5. Strengths and Weaknesses of the Nutrition Surveillance Systems in EMRO

The analysis of the different surveillance systems in the EMRO region showed several strong points. To start with, all countries have established surveillance systems that collect comprehensive data including anthropometric measures, biochemical data, and other relevant indicators to identify and track nutritional status, especially among vulnerable populations like pregnant women and children under 5 years. Moreover, these systems focus on micronutrient deficiency by collecting biochemical data, specifically hemoglobin and hematocrit levels, which help detect anemia and monitor micronutrient deficiencies, and as a result, provide valuable insights into the nutritional health of the target populations. Another strength point shown commonly in some countries such as Sudan, Syria, and Yemen, is that they collect clinical signs like edema, which can further enhance the accuracy of nutritional assessments. It is worth mentioning that all the included countries in this study recognize breastfeeding as an important indicator of child health and include it in their surveillance systems, highlighting the significance of optimal infant feeding practices. Concerning sampling techniques, several countries employ sampling methods to gather data, enabling them to estimate nutrition-related conditions at the population level. This helps in the efficient allocation of resources and the planning of further interventions.

There were also weaknesses in several points regarding the surveillance systems. One example is the variation in sampling design. The different sampling approaches used by countries, such as population projection, stratified cluster sampling, and cooperation with statistical bureaus, may introduce variability in the representativeness of the collected data. Another weakness is represented by incomplete geographic coverage. While stable settings aim for national coverage, systems operating in fragile settings have limited geographic coverage, potentially excluding some regions with high vulnerability. This can lead to gaps in the understanding of the nutritional status of the entire population. Additionally, the frequency of data collection varies significantly across countries, ranging from monthly to annually. Inconsistencies between the reporting period and data collection period can impact the timeliness and relevance of the reported information.

### 3.6. Threats and Opportunities

Some threats such as limited financial and human resources may hinder the expansion and improvement of the nutrition surveillance systems. Moreover, insufficient funding and staffing can lead to gaps in data collection, analysis, and reporting. Moreover, fragile political and social settings may hinder consistent surveillance activities due to political conflicts, social unrest, or disruptions in healthcare services. These conditions can impede data collection efforts and compromise the reliability of the reported information. Although many threats can affect the functionality and sustainability of surveillance systems, opportunities can be created, however, for improvement and enhancement. This can be through improved collaboration, where countries can collaborate and share best practices and success stories to improve the consistency and standardization of surveillance systems, leading to better comparability and increased data utility for regional and global analyses. Moreover, by integrating data from multiple sources, such as health facilities, surveys, and population censuses, countries can enhance the comprehensiveness and accuracy of their surveillance systems, providing a more holistic view of the nutritional situation.

## 4. Discussion

Nutrition surveillance aims to collect data on the nutrition status of the population to take responsive actions [7,8]. Obtaining strong and reliable data reflects the strength of nutrition surveillance and positively affects health and nutrition strategies and policies to prevent malnutrition [2,9]. Despite regional efforts by the World Health Organization (WHO), only a few countries in the EMRO region have established nutrition surveillance systems, while there is also a lack of published information on the designs of these systems and the challenges encountered during their development [2]. As a result, this study reviewed and compared the existing nutrition surveillance systems in the region. It examined the methods and frequency of data collection used by several countries and highlighted the challenges these systems face in terms of sustainability and optimal functionality.

In this study 8 out of 22 identified countries in the EMRO region participated in the study achieving a response rate of 38%. While it would have been ideal to have a higher response rate with more countries participating, the decision of whether or not to participate ultimately rests with each country’s authorities and stakeholders. Nevertheless, the data collected from the eight participating countries can still offer valuable insights into the nutrition surveillance systems in those specific nations and the findings and recommendations based on the data collected from the eight participating countries can be instrumental in shaping future efforts to enhance nutrition surveillance strategies in the EMRO region.

The findings of the study reveal that all countries that participated in the survey primarily rely on secondary data from health facilities to gather nutrition indicators regularly. Additionally, the study uncovered that certain countries, including Oman, Morocco, Palestine, Kuwait, and Sudan, adopt a more comprehensive approach by supplementing secondary data with primary data collected from sources such as school surveys or community surveys. This triangulation of data from multiple sources strengthens the reliability and accuracy of the information, as it allows for a more diverse and comprehensive assessment of the nutritional status of the population [10,11]. Furthermore, the study’s findings align with the results of a review conducted by Friedman et al., which also recognized Kuwait and Palestine for their practice of triangulating data collection from various sources. This congruence in findings adds credibility to the study’s results and reinforces the importance of adopting such comprehensive approaches to nutrition data collection and analysis [4]. On the other hand, Yemen and Syria, similar to the system in Somalia [4], primarily focus on nutrition surveillance systems that mainly screen children under five years in health facilities. However, they have the opportunity to complement their data with information from other resources, such as repeated SMART surveys and reports of health programs. The use of multiple data sources, such as combining secondary data from health facilities with primary data from school surveys or community surveys, can indeed provide a more holistic understanding of the nutrition landscape.

Another important finding from this study revealed significant variation in the frequency of nutrition surveys among countries in the region. This variation reflects the diversity in approaches to data collection and highlights the importance of tailoring surveillance efforts to meet each country’s specific context, resources, and priorities. Countries like Sudan, which conduct nutrition surveys every six months similar to Somalia [4,12], likely prioritize more frequent data collection due to their specific circumstances. Regular surveys every six months can provide timely and up-to-date information on the nutritional status of the population, especially in contexts where nutrition indicators might fluctuate rapidly due to factors like seasonal changes, conflicts, or other emergencies. On the other hand, countries like Palestine, Kuwait, and Saudi Arabia, conducting surveys annually, might find that this frequency adequately captures trends and changes in nutrition indicators over a longer period. Annual surveys can still provide valuable insights into the nutritional status and allow for the evaluation of nutrition-related interventions and policies. Morocco’s decision to conduct surveys every five years may be driven by factors such as resource constraints, the stability of nutrition indicators over longer periods, and the need to allocate resources to other pressing priorities. Five-year intervals could be considered appropriate when looking at more long-term trends and planning for policy and program development over a significant period. By adjusting the frequency of nutrition surveys, countries can strike a balance between the need for up-to-date information and the available resources. It also allows them to tailor data collection to their specific requirements for decision-making, planning, and monitoring nutritional trends effectively. Ultimately, the key is to ensure that the chosen survey frequency aligns with the country’s specific needs and the dynamic nature of nutrition challenges in the region. Regular and systematic data collection is vital for evidence-based decision-making and the development of effective interventions to improve the nutritional well-being of populations in the EMRO region.

After the World Food Conference in 1974, many countries, especially those facing emergencies, recognized the importance of establishing surveillance systems primarily for early warning purposes [13]. As revealed in this study, Yemen and Syria are examples of countries that have implemented nutrition surveillance systems with a strong focus on early warning capabilities. Early warning through nutrition surveillance is a powerful tool for rapid response to undernutrition and critical shortages of food in affected countries. By closely monitoring nutrition indicators and trends, these systems can help identify and predict emerging nutritional crises or deteriorating food security situations, prevent escalation, and enable better resource allocation [13,14].

The nutrition surveillance system that captures seasonal or monthly trends of malnutrition can enhance the early responses [15]. Nevertheless, modern nutrition surveillance systems have a broader focus in which they use integrated approaches including components that are seen to be comprehensive; additionally, these use nutrition-specific and nutrition-sensitive interventions to fight against malnutrition [16]. This focus came from the large shift in diets and their health implications and the different patterns of diets that are either loaded with saturated fat, sugars, and refined foods in some places or the increased famine in other places [17]. As a result, this study encourages enhancing the integrated approach, and thus nutrition indicators must fall under four basic categories: anthropometrics, biochemical data, food security, and nutrition transition. The first category of nutrition indicators focuses on anthropometrics, and our findings showed that all countries in the region adopt this component. Micronutrient deficiency is another component that also is common in most of the countries included in this study. However, one critical component for those countries suffering from certain emergencies, political instabilities, or conflict is food security surveillance. Although many countries in the region are facing difficult humanitarian situations, we found in this study that only Sudan includes food security indicators in the nutrition surveillance report using existing IPC surveys. The final component which is nutrition transition is a new component to address the risk factors for non-communicable diseases; most countries in EMRO with a stable setting have included some of these indicators such as measuring blood glucose levels and cholesterol levels.

It is also important to mention that the sustainability of the nutrition surveillance system is a critical issue that faces many countries in the world that initiated nutrition surveillance after the World Food Conference in 1974 [14]. Several factors hinder the sustainability of any nutrition surveillance system, such as inappropriate coordination within its structure, and lack of credibility of surveillance amongst stakeholders [9,14]. Moreover, the lack of sufficient funding and staff as shown in our results can lead to gaps in data collection, analysis, and reporting. Moreover, political conflicts, social unrest, or disruptions in healthcare services negatively contribute to the discontinuity and malfunction of these systems.

Most countries of stable political settings included in this study adopted lightweight structures by using the existing health information system to avoid extra cost. They succeeded in coordinating all other sources of information to fulfill the target of the nutrition surveillance system, many of which included information from national surveys such as in Palestine, Kuwait, Saudi Arabia, and Morocco. In contrast, countries facing conflict situations may rely on parallel systems due to the challenges posed by political instability and limited resources. These parallel systems, although driven by donations and external support, aim to collect data on nutrition situations amid challenging circumstances. Interestingly, Sudan’s ability to develop a nutrition information system despite political instability showcases resilience and adaptability in the face of challenging circumstances. By utilizing existing information and complementing it with periodic rapid community assessments, Sudan demonstrates a resourceful approach to collecting nutrition data. Accurate and comprehensive data are critical to provide reliable information to policymakers, program managers, and donors, to enhance the credibility of surveillance and the usage of information [15]. The quality of anthropometric data is also important in assessing how health and nutrition interventions are implemented and in guiding subsequent planning [16]. Therefore, most countries in the region adopted evaluation and monitory approaches to ensure the quality of their nutrition surveillance systems.

Despite the valuable findings of this study, it is important to acknowledge its limitations. One major limitation is the lack of assessment of the quality of nutrition surveillance reports. Evaluating the quality of these reports would have provided a deeper understanding of the accuracy and completeness of the data, as well as potential areas for improvement in data collection, analysis, and reporting processes. The low response rate from countries in the region is another limitation. While this may affect the overall representativeness of the study, the insights obtained from the participating countries still offer valuable contributions to understanding the nutrition surveillance landscape in the EMRO region. Despite the low response rate, the findings can serve as a starting point for further research and future studies. The scarcity of research funding, political instability, and conflict are additional challenges faced by some countries in the region. These factors can contribute to limited data availability and hinder comprehensive assessments of nutrition surveillance systems. The complex political situations in some countries may also affect the development and sustainability of these systems, and should be considered when interpreting the study’s results. Overall, being aware of these limitations allows for a more nuanced interpretation of the study’s findings and emphasizes the need for continued efforts in improving nutrition surveillance and data collection in the EMRO region. Addressing these limitations through further research, collaborations, and support can lead to more robust and effective nutrition surveillance systems, ultimately contributing to better health outcomes for populations in the region.

## 5. Conclusions

In conclusion, the findings of this study highlight the importance of integrating nutrition surveillance systems with existing health information systems to ensure sustainability, especially in fragile situations. Such integrated systems can provide a more sustained and cohesive approach to data collection and analysis. While community nutrition data add value and credibility to surveillance efforts, the implementation of community sentinel surveillance can be challenging in many countries in the region. Therefore, leveraging data from existing community surveys can be a more practical and feasible approach to enhance the comprehensiveness of nutrition surveillance. Furthermore, the study emphasizes the significance of having a structural framework for nutrition surveillance systems. A well-defined framework ensures the systematic collection, analysis, and reporting of nutrition indicators, enhancing the system’s effectiveness in monitoring nutritional trends and informing policy decisions. As a recommendation, a stepwise approach that includes essential nutritional components and a minimum set of indicators is proposed. This approach allows for a gradual and strategic expansion of the surveillance system’s capabilities, ensuring that it meets the specific needs and priorities of each country in the region. Overall, the study contributes valuable insights into the best practices and challenges faced by nutrition surveillance systems in the EMRO region. By adopting integrated and strategic approaches, countries can improve the functionality and impact of their surveillance efforts, leading to more informed decision-making and targeted interventions to address nutritional challenges effectively. These efforts are crucial in promoting better nutrition and well-being for populations in the region and beyond.

## Figures and Tables

**Table 1:** Data sources of nutrition surveillance used in different states in the Eastern Mediterranean Region.

Countries	Data Sources of Nutrition Surveillance				
	**Health Information** **System**	**Survey**	**Health Facility**	**Nutrition Center**	**Community and School**
Saudi Arabia		X	X		
Kuwait			X		X
Syria			X		
Yemen			X		
Oman	X	X	X		X
Morocco	X		X		X
Palestine			X		X
Sudan	X		X	X	X

X: available.

**Table 2:** The components of nutrition surveillance and the minimum indicators used in EMRO countries.

Surveillance Components	Indicators	Country
Anthropometrics	Wasting Stunting Overweight & Obesity Clinical signs: Edema	All 8 countries Clinical signs screening only in Sudan, Syria, and Yemen
Micronutrient	Biochemical: Hemoglobin, Ferritin, Iodine Program: Vitamin A, Folic acid Clinical signs: Neural Tube Defect	All 8 counties screen for anemia Palestine and Kuwait track Vitamin A Morocco screens for Neural Tube Defect
Food security	Food accessibility Food availability Food habits and intake Breastfeed	All 8 countries consider breastfeeding in nutrition surveillance but food security indicators are addressed only in Sudan. Palestine and Kuwait screen for dietary habits and food intake.
Nutrition transient	Cholesterol Blood glucose	Saudi Arabia

## Data Availability

Data can be shared upon request.

## References

[1] McGuire S. (2015). International Food Policy Research Institute. 2014. Washington, DC: Global Nutrition Report 2014: Actions and accountability to accelerate the world’s progress on nutrition. Adv. Nutr..

[2] Al Jawaldeh A., Osman D., Tawfik A. (2013). World Health Organization. Food and Nutrition Surveillance Systems: A Technical Guide for the Development of a Food and Nutrition Surveillance System for Countries in the Eastern Mediterranean Region.

[3] Farrag N.S., Cheskin L.J., Farag M.K. (2017). A systematic review of childhood obesity in the Middle East and North Africa (MENA) region: Prevalence and risk factors meta-analysis. Adv. Pediatr. Res..

[4] Friedman G. (2014). Review of National Nutrition Surveillance Systems.

[5] AlSumaie M. (2011). The Kuwait nutrition surveillance system (KNSS): An example for the region. First Regional Nutrition Conference-Nutrition Challenges in the East Mediterranean Region.

[6] WHO (2023). Interactive Map.

[7] Tuffrey V., Hall A. (2016). Methods of nutrition surveillance in low-income countries. Emerg. Themes Epidemiol..

[8] Tuffrey V. (2017). Nutrition Surveillance Systems: Their Use and Value.

[9] Maire B., Beghin I., Kolsteren P., Remaut-De Winter A. (2001). Nutrition Surveillance: A Sustainable Operational Approach.

[10] Carter N., Bryant-Lukosius D., DiCenso A., Blythe J., Neville A.J. (2014). The use of triangulation in qualitative research. Oncol. Nurs. Forum..

[11] Rutherford G.W., McFarland W., Spindler H., White K., Patel S.V., Aberle-Grasse J., Sabin K., Smith N., Taché S., Calleja-Garcia J.M. (2010). Public health triangulation: Approach and application to synthesizing data to understand national and local HIV epidemics. BMC Public Health.

[12] Food Security Analysis Unit for Somalia (2005). Nutrition: A Guide to Data Collection, Analysis, Interpretation, and Use.

[13] Harris E.W., Caballero B. (2013). Nutritional Surveillance: Developed Countries, in Encyclopedia of Human Nutrition.

[14] Tuffrey V. (2016). A perspective on the development and sustainability of nutrition surveillance in low-income countries. BMC Nutr..

[15] Micha R., Coates J., Leclercq C., Charrondiere U.R., Mozaffarian D. (2018). Global Dietary Surveillance: Data Gaps and Challenges. Food Nutr. Bull..

[16] European Centre for Disease Prevention and Control (2014). Data Quality Monitoring and Surveillance System Evaluation–A Handbook of Methods and Applications.

[17] Popkin B.M. (1998). The nutrition transition and its health implications in lower-income countries. Public Health Nutr..

